# Pharmacological Inhibition of the Vacuolar ATPase in Bloodstream-Form Trypanosoma brucei Rescues Genetic Knockdown of Mitochondrial Gene Expression

**DOI:** 10.1128/AAC.02268-17

**Published:** 2018-08-27

**Authors:** Claudia Schaffner-Barbero, Migla Miskinyte, Jaspreet Singh Grewal, Achim Schnaufer

**Affiliations:** a Institute of Immunology & Infection Research, University of Edinburgh, Edinburgh, United Kingdom; b Department of Biology, Centre for Immunology and Infection, University of York, York, United Kingdom

**Keywords:** F_1_F_o_-ATPase, RNA editing, Trypanosoma, V-ATPase, bafilomycin, kDNA, kinetoplast, mitochondria, vacuoles

## Abstract

Trypanosomatid parasites cause diseases in humans and livestock. It was reported that partial inhibition of the vacuolar ATPase (V-ATPase) affects the dependence of Trypanosoma brucei on its mitochondrial genome (kinetoplast DNA [kDNA]), a target of the antitrypanosomatid drug isometamidium. Here, we report that V-ATPase inhibition with bafilomycin A1 (BafA) provides partial resistance to genetic knockdown of mitochondrial gene expression. BafA does not promote long-term survival after kDNA loss, but in its presence, isometamidium causes less damage to kDNA.

## Abstract (precis)

Trypanosomatid parasites cause diseases in humans and livestock. It was reported that partial inhibition of the vacuolar ATPase (V-ATPase) affects the dependence of Trypanosoma brucei on its mitochondrial genome (kinetoplast DNA [kDNA]), a target of the antitrypanosomatid drug isometamidium.

## TEXT

Trypanosomatid parasites cause a range of debilitating or fatal diseases in humans and animals, typically transmitted by insect vectors (1, 2). Important diseases include Chagas disease, caused by Trypanosoma cruzi, various forms of leishmaniasis, caused by Leishmania spp., human African trypanosomiasis (also known as sleeping sickness), caused by T. brucei subspecies T. brucei gambiense and T. brucei rhodesiense, and the livestock disease nagana, caused by *T. vivax*, T. congolense, and, less frequently, T. brucei brucei.

Trypanosomatids belong to the clade Kinetoplastea, flagellated protists characterized by their unusual mitochondrial DNA, called the kinetoplast DNA (kDNA). T. brucei kDNA is a concatenated network of dozens of 23-kb maxicircles (the equivalent of mitochondrial DNA in other eukaryotes) and thousands of ∼1-kb minicircles that encode guide RNAs (gRNAs). The gRNAs guide posttranscriptional editing of most maxicircle-encoded mRNAs, a process that is essential for generating functional transcripts (3–5). Maintenance and expression of kDNA are essential in both the mammalian bloodstream form (BF) and the insect stage of T. brucei (3), and interference with kDNA maintenance is involved in the mode of action of some antitrypanosomatid drugs, such as ethidium bromide (EtBr) and isometamidium chloride (ISM) (6–8). However, BF T. brucei parasites appear to require only a single mitochondrial gene product for survival, subunit *a* of the F_o_ moiety of the F_1_F_o_-ATP synthase (although translation of the subunit *a* mRNA requires another kDNA-encoded protein, subunit RPS12 of the mitochondrial ribosome). In that stage of the life cycle, this complex operates in reverse, as an ATP-driven proton pump, to generate the mitochondrial membrane potential (9–11). Mutations in the nuclearly encoded γ-subunit of the ATP synthase, such as L262P, can fully compensate for the loss of kDNA in BF T. brucei (12) and result in a substantial decrease in ISM sensitivity (7, 13). The mechanism of compensation is not fully understood but appears to involve uncoupling of F_1_ from F_o_ and altered kinetics (11, 12).

Recently, it was reported that perturbation of the vacuolar ATPase (V-ATPase) affects mitochondrial ATPase function and kDNA dependence in trypanosomes. V-ATPase is essential in T. brucei, but sublethal inhibition of the complex by low-efficiency RNAi or with the V-ATPase inhibitor bafilomycin A1 (BafA) permitted survival for at least 3 days in the presence of normally lethal concentrations of ISM (14). The physiological mechanism of compensation remained obscure. We decided to investigate the effects of sublethal concentrations of BafA on kDNA dependence over longer time scales. All methods were performed as described previously (7, 12), unless otherwise specified.

We first investigated whether V-ATPase inhibition affects the dependence of BF T. brucei on RNA editing. RNA editing ligase 1 (REL1) is a key component of the T. brucei editosome, and its knockdown is lethal (15, 16). Expression of an ATP synthase γ-subunit with an L262P mutation fully rescues from this phenotype (12). If partial inhibition of the V-ATPase by BafA renders cells impervious to kDNA loss, treatment with the drug should also rescue from the growth phenotype observed upon knockdown of REL1.

**FIG 1:**
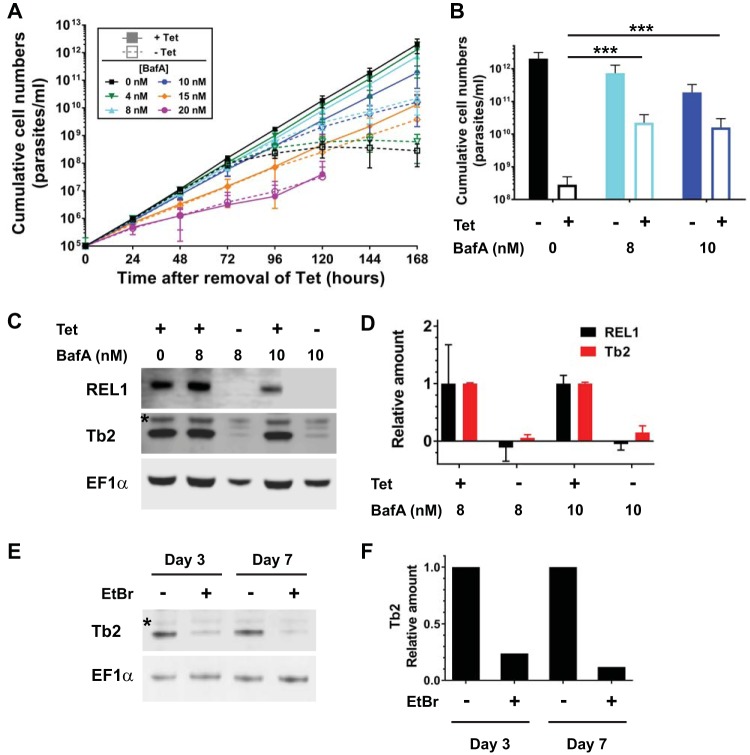
(A) Cumulative growth curves of T. brucei REL1-cKO BF cells cultured in the presence (filled symbols, solid lines) and absence (open symbols, dashed lines) of 1 μg/ml tetracycline (Tet; required for expression of REL1) and at various concentrations of BafA. Each data point is the average of at least six separate growth curves; error bars indicate the standard deviation (SD). (B) Comparison of cumulative cell numbers (A) after 168 h at 0 nM (*n* = 6), 8 nM (*n* = 8), and 10 nM (*n* = 6) BafA. Statistical significance of differences was assessed with the Wilcoxon rank sum test; *P* < 0.001 (***) was for noninduced (−Tet) 0 nM BafA versus −Tet 8 nM BafA and versus −Tet 10 nM BafA. (C) Western blot of samples taken at 0, 8, and 10 nM BafA after 168 h, probed with a REL1 antibody. The same blot was probed with antibodies for Tb2, to assess levels of intact F_1_F_o_-ATPase complex (the asterisk indicates a cross-reacting protein), and for EF-1α (Millipore), as a loading control. (D) Quantification of Western blot signals, taking the average of two replicates (one shown in panel C) and indicating relative protein levels under noninduced compared to induced (+Tet) conditions for each BafA concentration (normalized to EF-1α). (E) Western blot of samples from T. brucei BF cells expressing an ATPase subunit-γ allele with the L262P mutation, taken after 3 and 7 days of culturing in the presence of 10 nM EtBr to remove kDNA. Cells grown in the absence of EtBr were used as controls. The blot was probed with antibodies for F_1_F_o_-ATPase subunit Tb2 (the asterisk indicates a cross-reacting protein; see panel C) and for EF-1α as loading control. (F) Relative quantification of the Tb2 Western blot signals shown in panel E (normalized to EF-1α).

We used a REL1 conditional knockout cell line (REL1-cKO), where an ectopic copy of the REL1 gene is under the control of a tetracycline (Tet)-inducible promoter and both endogenous REL1 alleles have been deleted (15). After the removal of Tet from the medium, cKO-REL1 cells exhibited a rapid and severe growth defect, with growth ceasing completely after 96 h (Fig. 1A, dashed black curve), and no live cells being visible under the microscope at later time points, as observed before (15). The presence of 8 nM or 10 nM BafA alleviated the growth defect, with cells continuing to proliferate 168 h after Tet removal (Fig. 1A and B, dashed cyan and blue curves and columns, respectively) despite REL1 being below the detection limit in a Western blot assay (Fig. 1C and D; all image acquisitions and analyses were performed digitally with Li-Cor Odyssey or C-DiGit systems). Lower concentrations of BafA did not alleviate the growth defect caused by REL1 depletion (Fig. 1A, green curves), while higher BafA concentrations caused a severe growth defect even in the presence of Tet (Fig. 1A, pink curves). We note that the range of concentrations in which rescue occurred was narrow and varied slightly between experiments and BafA stocks (data not shown). To investigate if BafA affected the knockdown of RNA editing itself, we assessed levels of the F_1_F_o_-ATPase subunit Tb2. The stability of this protein depends on presence of the kDNA-encoded F_o_ subunit *a*, in that in the absence of functional kDNA (and thus subunit *a*), the level of Tb2 is substantially reduced (Fig. 1E and F) (17). We found that even in the presence of 8 nM or 10 nM BafA, knockdown of REL1 reduced the levels of Tb2 to at least the same extend as with the loss of kDNA (Fig. 1C). We conclude that the rescue effect provided by BafA appears to reduce dependence on a functional F_1_F_o_-ATPase, as suggested by Baker et al. (14).

**FIG 2:**
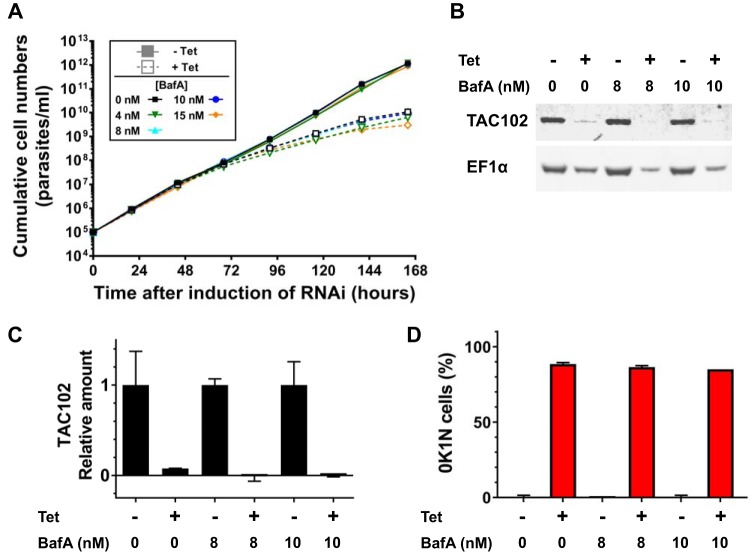
(A) Cumulative growth curves of T. brucei TAC102 RNAi BF cells cultured in the absence (filled symbols, solid lines) and presence (open symbols, dashed lines) of 1 μg/ml Tet and at various concentrations of BafA. Each data point is the average of 2 growth curves; error bars indicate the SD. (B) Western blot of samples taken at 0, 8, and 10 nM BafA after 168 h, probed with antibodies for TAC102 and EF-1α. (C) Quantification of Western blot signals, taking the average of two replicates (one shown in panel B) and indicating relative protein levels under RNAi-induced (+Tet) to noninduced (−Tet) conditions for each BafA concentration (normalized to EF-1α). (D) Loss of kDNA (0K1N cells) assessed by DAPI staining and microscopy after 168 h of culturing.

Next, we investigated if treatment with BafA would rescue from cell death caused by kDNA loss, induced either genetically or pharmacologically. To induce kDNA loss genetically, we repressed expression of the TAC102, an essential component of the tripartite attachment complex and thus required for kDNA segregation during cell division (18). As expected, Tet-induced TAC102 knockdown in the published RNAi cell line (18) caused a severe growth defect and kDNA loss (Fig. 2). In contrast to the RNA editing knockdown experiment, however, we did not observe any rescue of either phenotype by incubation with BafA (Fig. 2A and D).

**FIG 3:**
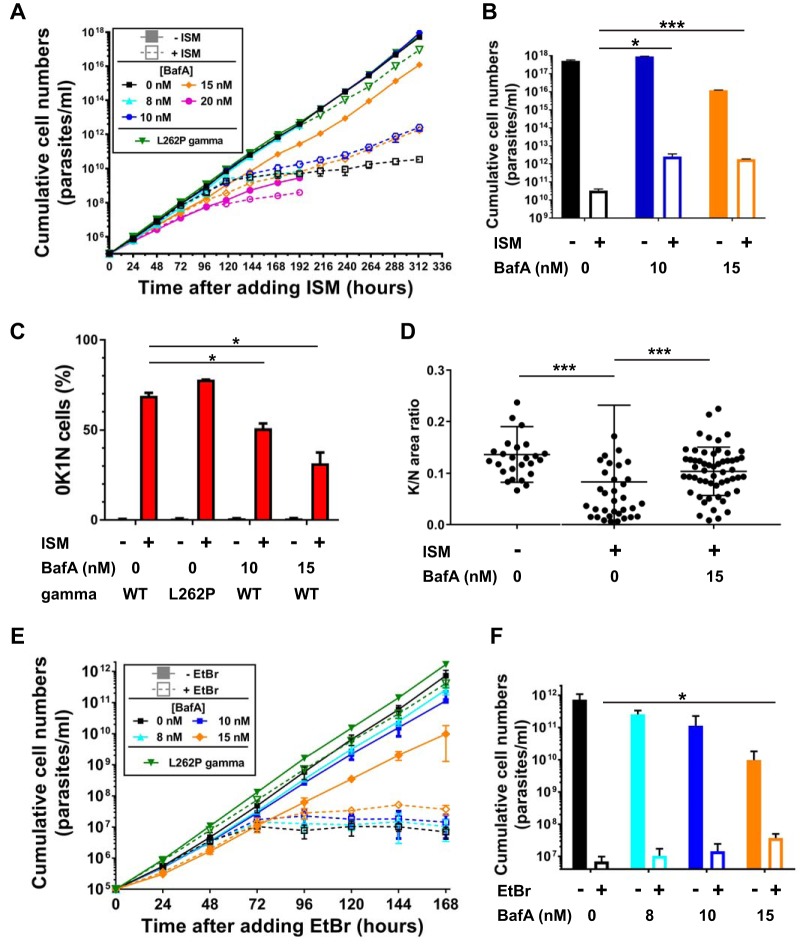
(A) Cumulative growth curves for T. brucei BF cells cultured in the absence (filled symbols, solid lines) and presence (open symbols, dashed lines) of 0.1 nM ISM and at various concentrations of BafA. In parallel, a cell line expressing an ATPase subunit-γ allele with the L262P mutation was also grown in the absence of presence of 0.1 nM ISM (green triangles and lines). Each data point is the average of two growth curves; error bars indicate the SD. (B) Comparison of cumulative cell numbers (in panel A) after 313 h. Statistical significance of differences was assessed with the Student unpaired *t* test; 0.01 < *P* < 0.05 (*) was for 0 nM BafA +ISM versus 10 nM BafA +ISM; *P* < 0.001 (***) was for 0 nM BafA +ISM versus 15 nM BafA +ISM. (C) Loss of kDNA (0K1N cells) assessed by DAPI staining and microscopy after 96 h of culturing. Statistical significance of differences was assessed with the Student unpaired *t* test; 0.01 < *P* < 0.05 (*) was for the wild type (WT) +ISM 0 nM BafA versus WT +ISM 10 nM BafA or versus WT +ISM 15 nM BafA. (D) The relative amount of kDNA in 1K1N cells after 96 h of culturing was assessed by DAPI staining and quantitation of kinetoplast versus nucleus fluorescence intensity. Statistical significance of differences was assessed with the Mann-Whitney test; *P* < 0.001 (***) for −ISM 0 nM BafA (*n* = 26) versus +ISM 0 nM BafA (*n* = 35); *P* < 0.001 (***) for +ISM 0 nM BafA versus +ISM 15 nM BafA (*n* = 56). (E) Cumulative growth curves of T. brucei BF cells cultured in the absence (filled symbols, solid lines) and presence (open symbols, dashed lines) of 10 nM EtBr and at various concentrations of BafA. In parallel, a cell line expressing an ATPase subunit-γ allele with the L262P mutation was also grown in the absence of presence of 10 nM EtBr (green triangles and lines). Each data point is the average from four separate growth curves; error bars indicate the SD. (F) Comparison of cumulative cell numbers (in panel E) after 168 h. Statistical significance of differences was assessed with the Wilcoxon rank sum test; 0.01 < *P* < 0.05 (*) was for +EtBr 0 nM BafA versus +EtBr 15 nM BafA.

To induce kDNA loss pharmacologically, we treated BF T. brucei cells with 0.1 nM ISM. At this concentration, ISM causes kDNA loss but only a very minor growth defect in kDNA-independent cells, as demonstrated by transgenic expression of an F_1_ γ-subunit allele with the L262P mutation (Fig. 3A, green triangles and lines; see also reference 7). In the absence of BafA, kDNA-dependent cells showed a severe growth defect, in that cells complete stopped proliferating after 168 h (Fig. 3A, black squares and lines), with few, if any, surviving cells visible by microscopic inspection after 240 h or later. In contrast, in the presence of 10 nM or 15 nM BafA, cells continued to proliferate, albeit at much lower rates (Fig. 3A, blue and orange symbols and lines). The difference in cumulative growth after 313 h (the endpoint of the experiment) was reproducible and statistically significant (Fig. 3B). At 8 nM BafA, the rescue effect was less pronounced (Fig. 3A, cyan triangles and lines, experiment terminated after 192 h), while 20 nM BafA caused a severe growth defect even in the absence of ISM, as observed before (Fig. 3A and 1A, pink circles and lines, experiment also terminated after 192 h). In contrast to what we had found for genetic induction of kDNA loss, we observed that BafA afforded significant protection from kDNA loss caused by ISM. After 96 h of exposure to ISM, about 70% of the control cells had lost their kDNA, as observed by staining with 4′,6-diamidino-2-phenylindole (DAPI) and microscopy (Fig. 3C; 0K1N, cells with 1 nucleus but no kinetoplast). In cultures where we added 10 nM or 15 nM BafA to the growth medium, the fractions of 0K1N cells present at 96 h were reduced to ∼50% and ∼30%, respectively (Fig. 3C). In cells where a kinetoplast was visible (1K1N cells), quantitation of kDNA by measuring relative fluorescence compared to the nucleus confirmed that exposure to ISM for 96 h caused a significant reduction in the amount of organellar DNA, presumably an intermediate stage to complete kDNA loss (Fig. 3D). Incubation with 15 nM BafA ISM provided highly significant protection from this effect (Fig. 3D). Taken together, the experiments with kDNA loss induced genetically or by ISM treatment suggest the following: pharmacological inhibition of the V-ATPase with BafA cannot protect T. brucei from the lethal effects of complete loss of kDNA. Instead, BafA reduces kDNA damage and loss caused by ISM.

Finally, we induced kDNA loss with an alternative pharmacological regime, exposure to 10 nM EtBr. Like ISM, EtBr is a compound from the phenanthridine class that at low concentrations causes kDNA loss but affects growth of kDNA-independent cells only mildly (Fig. 3E, green triangles and lines represent growth of cells expressing a γ-subunit with the L262P mutation; see also references 7 and 8). We found that simultaneous incubation with 15 nM BafA resulted in a reproducible rescue from cell death caused by treatment with EtBr (Fig. 3E and F), although the effect was much less pronounced than with ISM, and very few surviving cells were apparent after 168 h (the endpoint of this experiment).

These results pose two important questions. First, why did we observe robust rescue by BafA of growth defects caused by knockdown of RNA editing but not of growth defects caused by inhibition of kDNA maintenance? To our present knowledge, the two processes serve the same goal in BF T. brucei, namely, the production of F_1_F_o_ subunit *a* (12). One potential explanation is that partial inhibition of V-ATPase makes the parasites less dependent on proton pumping by the F_1_F_o_-ATPase, for example, by changing the pH balance between cellular compartments, but it does not make the parasites completely independent from it. A similar scenario has been proposed for yeast cells lacking mitochondrial DNA (19). The inducible gene expression system that we used in our study to knock down REL1 is unlikely to achieve complete repression in the absence of inducer (20), presumably resulting in some residual expression of REL1 and, consequently, subunit *a*. In contrast, interference with kDNA maintenance will result in an “all or nothing” response in affected cells, i.e., cells that lose kDNA will completely lose subunit *a* and F_1_F_o_-ATPase proton-pumping activity. We must note, however, that REL1 in repressed cells was below the detection limit of our Western blot analysis, and F_o_ subunit Tb2 in these cells was reduced to levels comparable to what we observe in cells depleted of kDNA. Testing this hypothesis will require the development of more sensitive and highly quantitative methods for the detection of intact F_1_F_o_-ATPase. Second, why was BafA more effective in rescuing the cells from the effects of ISM than the related EtBr? Our results provide indirect evidence that V-ATPase inhibition by BafA reduces mitochondrial uptake of ISM. How ISM and EtBr enter the mitochondrion is unknown, although efficient uptake of at least ISM appears to depend on the mitochondrial membrane potential (13). Our results suggest that the mitochondrial uptake mechanisms for ISM and EtBr may not be identical and that V-ATPase inhibition affects uptake of ISM more than uptake of EtBr. Resolving this question will require the development of methods to specifically measure the mitochondrial uptake of these compounds.

In summary, our results indicate complex effects of sublethal inhibition of the V-ATPase on T. brucei that affect both the degree of dependence on kDNA-encoded products as well as mitochondrial uptake of kDNA-intercalating trypanocides. Our study corroborates an intriguing link between vacuolar and mitochondrial ATPase function that begs further investigation. Furthermore, we suggest that pharmacological inhibition of V-ATPase function can be a useful research tool in the study of otherwise lethal perturbations of mitochondrial gene expression in BF T. brucei, but it should be noted that any experiment will require careful titration of inhibitors.
